# Effects of intranasal oxytocin on fear extinction learning

**DOI:** 10.1038/s41386-024-01996-y

**Published:** 2024-09-24

**Authors:** Mahmoud Rashidi, Joe J. Simon, Katja Bertsch, Gerhard Vincent Wegen, Beate Ditzen, Herta Flor, Valery Grinevich, Robert Christian Wolf, Sabine C. Herpertz

**Affiliations:** 1 Department of General Psychiatry, Center for Psychosocial Medicine, Heidelberg University, Heidelberg, Germany; 2 Department of Psychiatry and Behavioral Health, Wexner Medical Center, The Ohio State University, Columbus, OH USA; 3 Department of General Internal Medicine and Psychosomatics, Center for Psychosocial Medicine, Heidelberg University, Heidelberg, Germany; 4 Department of Psychology, Julius Maximilians University of Wuerzburg, Wuerzburg, Germany; 5 Institute of Medical Psychology, Center for Psychosocial Medicine, Heidelberg University, Heidelberg, Germany; 6 Institute of Cognitive and Clinical Neuroscience, Central Institute of Mental Health, Medical Faculty Mannheim, Heidelberg University, Mannheim, Germany; 7 Department of Neuropeptide Research in Psychiatry, Central Institute of Mental Health, Medical Faculty Mannheim, Heidelberg University, Mannheim, Germany; 8 Center for Neuroinflammation and Cardiometabolic Diseases, Georgia State University, Atlanta, GA USA; 9 DZPG, German Center for Mental Health, Berlin, Germany

**Keywords:** Translational research, Trauma, Fear conditioning

## Abstract

Once a threat no longer exists, extinction of conditioned fear becomes adaptive in order to reduce allotted resources towards cues that no longer predict the threat. In anxiety and stress disorders, fear extinction learning may be affected. Animal findings suggest that the administration of oxytocin (OT) modulates extinction learning in a timepoint-dependent manner, facilitating extinction when administered prior to fear conditioning, but impairing it when administered prior to extinction learning. The aim of the present study was to examine if these findings translate into human research. Using a randomized, double-blind, placebo-controlled, 2-day fear conditioning and extinction learning design, behavioral (self-reported anxiety), physiological (skin conductance response), neuronal (task-based and resting-state functional magnetic resonance imaging), and hormonal (cortisol) data were collected from 124 naturally cycling (taking no hormonal contraceptives) healthy females. When administered prior to conditioning (Day 1), OT, similar to rodent findings, did not affect fear conditioning, but modulated the intrinsic functional connectivity of the anterior insula immediately after fear conditioning. In contrast to animal findings, OT impaired, not facilitated, extinction learning on the next day and increased anterior insula activity. When administered prior to extinction learning (day 2), OT increased the activity in the bilateral middle temporal gyrus, and similar to animal findings, reduced extinction learning. The current findings suggest that intranasal OT impedes fear extinction learning in humans regardless of the timepoint of administration, providing new insights and directions for future translational research and clinical applications.

## Introduction

Once a threat no longer exists, it is generally adaptive to extinguish fear responses towards cues associated with that threat [1–3]. Pavlovian conditioning has been utilized extensively in animal [4] and human [5] studies to investigate fear extinction learning. During conditioning, a neutral conditioned stimulus (CS; e.g., a tone or light flash) is paired with an aversive unconditioned stimulus (US; e.g., a painful stimulus) and the CS elicits a fear response via repeated CS-US co-exposure. During extinction, the US no longer occurs and the fear response to the CS attenuates. It is widely accepted that extinction learning creates a new association (CS without US) that competes with and inhibits the initial association (CS and US) [4].

Extinction deficits might be involved in anxiety and stress-related disorders including phobias, panic disorder as well as post-traumatic stress disorder [6, 7]. Psychological (e.g., exposure-based strategies) and pharmacological interventions have been used to understand the mechanisms underlying extinction learning and subsequently provide mechanism-based treatments. Oxytocin (OT) is known to play a role in modulating the neural circuits implicated in fear extinction [8–11]. In a seminal rodent study [12], it was found that while the injection of OT into the brain ventricles prior to conditioning did not affect fear conditioning, it exerted anxiolytic effects and facilitated fear extinction during extinction learning on the next day. In contrast, administration of OT prior to extinction learning exerted anxiogenic effects and impaired fear extinction. The animal results suggest that the effects of OT on fear processes vary depending on the timepoint of its administration.

It is still largely unknown whether OT effects on fear extinction in humans are also timepoint-dependent. Unlike animal studies, most human studies have not conducted fear conditioning and extinction on separate days. The separation of these phases permits the examination of the effects of OT on each phase more accurately and independently. Moreover, fear memory consolidation requires time [13, 14] and neural circuit switching [15], and therefore single-session experiments are not optimal. Furthermore, most human studies have applied intranasal OT prior to phases related to the extinction process (e.g., extinction learning or extinction recall) rather than the initial fear acquisition phase [16–18], and thus the effects of the phase of OT application on various aspects of fear learning are largely unexplored. See Table 1, for a summary of some human studies on fear extinction learning.

**Table 1:** The effects of intranasal oxytocin (OT) on fear extinction learning.

Authors	N	% male	Time of OT administration	Dose (IU)	Phase			Intranasal OT effects
					Day 1	Day 2	Day 3	
Acheson et al. [42]	44	52	After fear conditioning but prior to extinction learning	24	Fear conditioning Extinction learning	Fear recall		Increased fear response in the early phase; no effects in the late phase
Eckstein et al. [43]	62	100	After fear conditioning but prior to extinction learning	24	Fear conditioning Extinction learning			Increased fear response in the early phase; decreased fear response in the late phase
Hu et al. [44]	61	47	After fear retrieval or at no fear retrieval	40	Fear conditioning	Fear retrieval or No fear retrieval	Extinction learning Reinstatement	No effects in the early phase; decreased fear response in the late phase only if fear retrieval conducted on day 2

Only healthy participants were recruited in these studies.

*CS* conditioned stimulus.

The aim of the present study was to examine if the rodent findings on timepoint-dependent OT effects on fear extinction learning translate into human research. A two-day design was employed to allow time between fear conditioning and fear extinction. We hypothesized that the administration of intranasal OT on day 1 prior to fear conditioning would not affect fear conditioning, but would modulate fear extinction on day 2. We anticipated that intranasal OT would affect fear memory consolidation measured by resting-state functional magnetic resonance imaging (fMRI) immediately after fear conditioning. We expected that intranasal OT administration on day 2 prior to extinction learning would modulate fear extinction. As intranasal OT reaches both the general blood circulation and the brain [19] and given the complex interactions between peripheral and central OT [9, 20, 21], we made no specific predictions about the direction (facilitation or impairment) of the effects.

## Methods and materials

### Participants

One-hundred twenty-four right-handed, non-smoking, premenopausal, non-pregnant, non-lactating, naturally cycling (taking no hormonal contraceptives) females without neurological or psychiatric disorders aged between 18 and 46 years (mean ± standard deviation: 24.43 ± 5.20) participated in this randomized, double-blind, placebo-controlled, two-day fMRI study. Participants were tested in the luteal phase of their cycle. The cycle phase was validated by self-report and blood assays (estradiol, progesterone). The procedure was explained to the participants and informed written consent was obtained. Participants attended the study over two consecutive days at the same time of the day to control for circadian variation. They were asked not to eat, drink, or brush their teeth at least one hour prior to the experiment. They received 80 euros for their participation. The study was approved by the Ethics Committee of Heidelberg University, and experimental procedures conformed to the Declaration of Helsinki.

### Experimental procedure

**Fig. 1:**
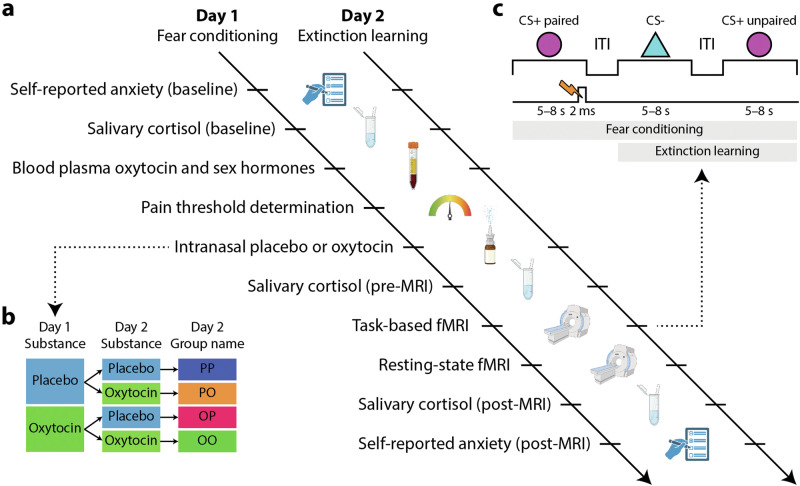
Schematic illustration of experimental procedure. **a** Fear conditioning took place on day 1 and extinction learning on day 2 at the same time of the day. Self-reports, salivary, and blood samples were collected at multiple time points. **b** Participants received either intranasal placebo or oxytocin (OT) on day 1 as well as day 2, resulting in two groups on day 1 and four groups on day 2. **c** On day 1 (fear conditioning), a circle was presented for 5–8 s and co-terminated with a short (2 ms) electric stimulus in 12 out of 16 trials (CS+ paired) and without an electric stimulus in 4 other trials (CS+ unpaired). A triangle was presented 16 times, and it was never paired with an electric stimulus (CS−). On day 2 (fear extinction), CS− and CS+ unpaired trials were each presented 16 times. No electric stimulus was applied on day 2. CS+ (paired and unpaired) and CS− trials were randomly interleaved. The geometric shapes (circle or triangle) associated with CS− and CS+ were counterbalanced across subjects. CS conditioned stimulus, ITI inter-trial interval.

Self-reported anxiety, salivary cortisol, and blood samples were collected at different time points before and after fear conditioning (day 1) and extinction learning (day 2). For details on the experimental procedure, see Fig. 1. The pain threshold was determined on day 1 for each participant to be of intensity 5 on a 0–10 scale (see Supplementary Materials for details). Afterwards, participants were randomly assigned either to the placebo or OT groups and received intranasal placebo or OT (24 IU; Syntocinon; Novartis, Switzerland; six puffs per nostril with an inter-puff interval of 30 s). For details on randomization of participants into groups, see Fig. 1b and the CONSORT flowchart in Supplementary Fig. 1. ~45 min after substance administration, participants performed the fear conditioning task on day 1 and extinction learning on Day 2, both inside the MRI scanner. Resting-state fMRI was collected immediately thereafter on both days.

### Experimental paradigm

During fear conditioning (day 1), a circle was shown in the middle of a screen for 5–8 s (mean = 6.5 s) which co-terminated with a brief (2 ms) painful electric stimulus (US) in 12 trials (CS+ paired) and without electric stimulus in 4 trials (CS+ unpaired; see Fig. 1c). A triangle was presented in the middle of the screen for 5–8 s and none of the 16 trials was co-terminated with the electric stimulus (CS−). An inter-trial interval of 15–20 s (mean = 17.5 s) was used. The geometric shape associated with CS− and CS+ was counterbalanced across subjects. A habituation phase was used on day 1 prior to fear conditioning and consisted of one CS+ unpaired and one CS− trial. Extinction learning (day 2) was similar to the conditioning phase except that no electric stimulus was applied. The electrodes for electric stimulation were nevertheless placed on the participant’s right forearm.

### Data acquisition and preprocessing

The details on the acquisition and preprocessing of behavioral (self-reported current anxiety levels), physiological (skin conductance response; SCR), neuronal (task-basked and resting-state fMRI), and hormonal (endogenous OT, cortisol, estradiol, progesterone, and testosterone) measures are provided in Supplementary Materials. Endogenous OT was collected to check no baseline differences existed across groups. Sex hormones (estradiol, progesterone, and testosterone) were collected to validate the phase of the menstrual cycle.

### Statistical analysis

#### Behavior

For day 1, a two-tailed independent-samples *t* test was used to compare the total scores for STAI-state. For day 2, a one-way ANOVA was used separately for baseline and post-MRI to compare total STAI-state scores. To compare the means of the memory of pain intensity and unpleasantness across the placebo and OT groups, two-tailed independent-sample *t* tests were used.

#### Psychophysiology

For fear conditioning, the mean SCRs of the placebo and OT groups were compared using a two-tailed independent-samples *t* test. For extinction learning, a one-way ANOVA was used for between-subject comparison of SCRs.

#### Task-based fMRI

Fear conditioning and extinction learning were modeled separately. In the first-level analysis, event onsets (CS− and CS+) with a duration of 5–8 s depending on the length of CS were modeled as boxcar functions. Note that since the electric stimulus in the conditioning phase occurred at the end of CS+ paired trials, it did not overlap with the selected time windows and thus the brain activities relate to the anticipation of pain rather than pain itself. For fear conditioning, all 16 CS− and 16 CS+ trials were included. For extinction learning, the first two CS− and CS+ trials were omitted to allow for learning that the electric stimulus may no longer occur. The design matrix contained two regressors modeling the CS− and CS+ conditions against the baseline convolved with a hemodynamic response function and a constant term. Contrast images were created by subtracting CS− from CS+. In the second-level analysis, contrast images created in the first-level analysis were compared across groups using independent-sample *t* tests and one-way ANOVA for the fear conditioning and extinction learning, respectively. For exploring the association between brain activations and physiological measures (SCR), multiple regression was used. Clusters were family-wise error (FWE) corrected for multiple comparisons at *p* < 0.05 with cluster-forming threshold at *p* < 0.001, uncorrected, with minimum ten contiguous voxels.

#### Resting-state fMRI

In the first-level analysis, a whole-brain seed-to-voxel analysis was carried out using the left anterior insula as a seed. (This region was selected as first) during extinction learning, a significant hyperactivation was observed in the OT group compared to the placebo group. (See results on extinction learning for further details; and second) The anterior insula has consistently been reported to be involved in extinction learning [5]. See Supplementary Materials for a detailed description of the procedure.

#### Hormones

Baseline hormonal levels (endogenous OT, estradiol, progesterone, and testosterone in blood plasma as well as salivary cortisol) were compared between groups (placebo or OT) using two-tailed independent-samples *t* tests, and between days (day 1 or day 2) using two-tailed paired-samples *t* tests (Supplementary Table 3). One-way ANOVAs were used to assess the effects of group on salivary cortisol on day 2 for baseline, pre-, and post-MRI measurements. Log transformation was employed to establish an approximately normal distribution before analysis of cortisol data.

## Results

### Fear conditioning (day 1)

#### Behavior

**Fig. 2:**
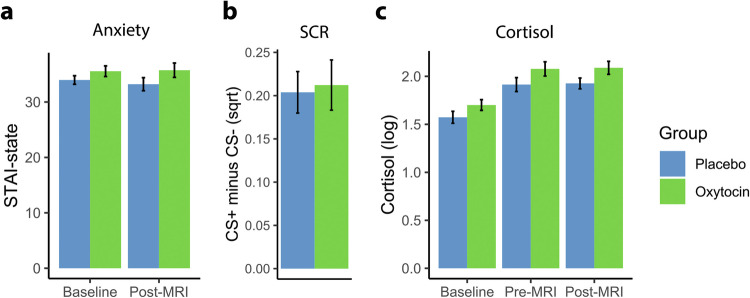
Effects of intranasal oxytocin (OT) on fear conditioning. **a** State anxiety levels of the placebo and OT groups were not significantly different at baseline or post-MRI. **b** Skin conductance responses (SCRs) were not significantly different across groups. **c** Cortisol concentrations in saliva were not significantly different between groups at baseline, pre-MRI, or post-MRI. These results suggest that intranasal OT did not modulate fear acquisition.

Baseline state anxiety did not differ between the placebo (33.96 ± 5.84) and OT groups (35.55 ± 7.23), *t*(112) = −1.29, *p* = 0.20, suggesting that participants of both groups showed similar baseline anxiety levels. Also, there was no significant difference for post-experiment state anxiety between the placebo (33.31 ± 8.88) and OT (35.73 ± 9.77) groups, *t*(112) = −1.44, *p* = 0.15, indicating that intranasal OT did not modulate self-reported anxiety measured after fear conditioning (see Fig. 2a).

#### Psychophysiology

The task was effective to evoke conditioned fear towards the CS+ compared to CS− stimuli. A significantly larger amplitude for CS+ trials (0.19 ± 0.22) compared to CS− trials (−0.02 ± 0.08) was observed, *t*(98) = −11.20, *p* < 0.001, *d* = 1.12 (see Supplementary Fig. 2 for the average amplitude of each trial). It should be noted that the time window within which the SCR was calculated was prior to the occurrence of the electric stimulus, thus the SCR reflecting the anticipatory responses to pain (pain-related fear), rather than the pain itself. No significantly different SCR was observed between the placebo (0.20 ± 0.17) and OT (0.21 ± 0.20) groups, *t*(97) = −0.22, *p* = 0.82, resembling the observations in the previous rodent study [12] that found no effects of OT on freezing behavior during fear acquisition (see Fig. 2b). For visualization and analysis of the early and late phase of extinction learning, see Supplementary Fig. 3.

#### fMRI

To examine whether the experimental task successfully activated the brain regions involved in fear acquisition, the differential BOLD responses of CS+ compared to CS− for all participants (placebo and OT groups combined) were tested. Known regions from a previous review [22] were observed including the anterior insular cortex extending to the frontal operculum, the ventral striatum including the putamen, a large expanse of the medial wall cortex including the anterior cingulate gyrus, the dorsolateral prefrontal cortex, and the lateral cerebellum. See Supplementary Fig. 4 for anatomical localization and Supplementary Table 4 for statistical and stereotaxic details. Regarding differences between placebo and OT groups, whole-brain analysis revealed no significant BOLD signal for the differential contrast of CS+ versus CS− (see Supplementary Table 5 for details). This result suggests that intranasal OT did not significantly modulate the BOLD responses during fear acquisition.

#### Hormones

Baseline hormones including OT, estradiol, progesterone, testosterone from blood plasma as well as cortisol from saliva samples were not significantly different between the placebo and OT groups (all *p*s > 0.13), suggesting that the participants in both groups had similar hormonal profiles. For details, see Supplementary Table 2. The pre-MRI (placebo: 1.91 ± 0.55; OT: 2.08 ± 0.55; *t*(110) = −1.57, *p* = 0.119) and post-MRI (placebo: 1.93 ± 0.42; OT: 2.09 ± 0.50, *t*(110) = −1.86, *p* = 0.067) cortisol levels between the placebo and OT groups were not significant although a trend towards significance emerged at post-MRI with higher cortisol levels in the OT group (see Fig. 2c).

### Resting-state fMRI (day 1)

**Fig. 3:**
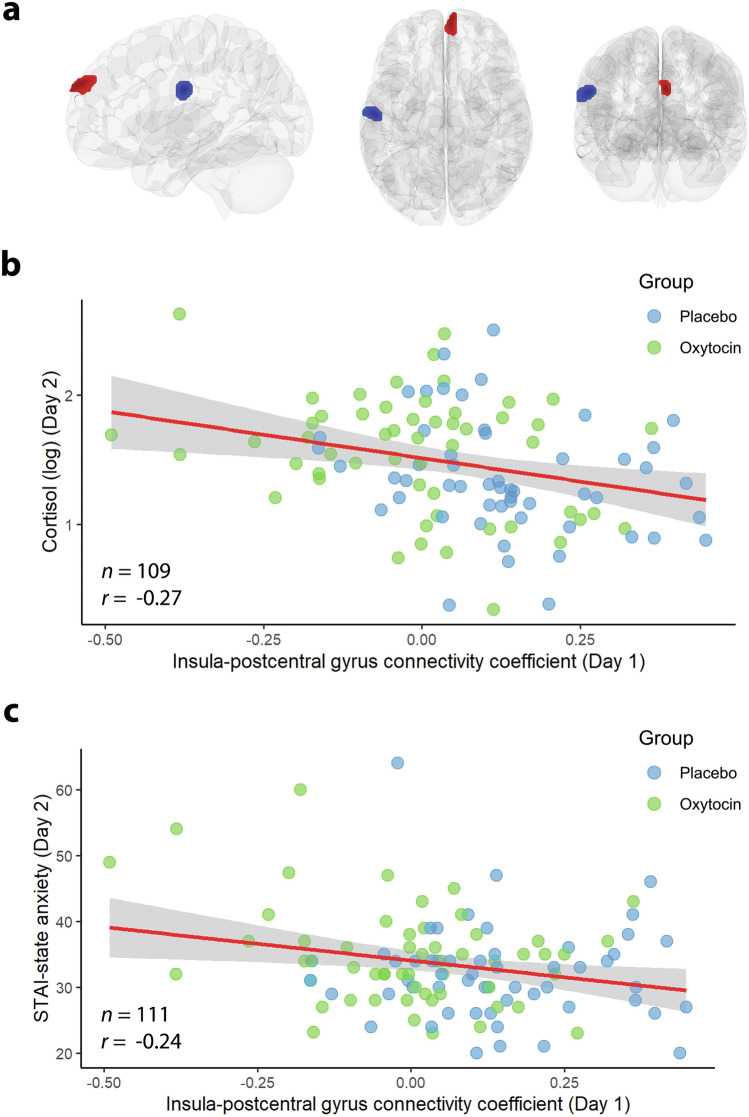
Effects of intranasal oxytocin (OT) on fear memory consolidation following fear conditioning. **a** Seed-to-voxel resting-state functional connectivity after fear conditioning revealed that in the OT compared to the placebo group the left anterior insula showed a stronger negative correlation with the left postcentral gyrus (blue bulb) as well as a stronger positive correlation with the right ventromedial prefrontal cortex (vmPFC) and the right superior frontal gyrus (red bulb). **b** Significant negative correlation between the anterior insula-postcentral gyrus connectivity and baseline cortisol levels on the next day prior to extinction learning. **c** Significant negative correlation between the anterior insula-postcentral gyrus connectivity and the baseline state anxiety on day 2 prior to extinction learning. The red line represents the fitted line and the gray areas are the confidence intervals at 95%.

The connectivity of the anterior insula was altered in the OT group, compared to the placebo group. Specifically, seed-to-voxel analysis revealed a stronger negative connectivity of the left anterior insula with an area predominantly (99%) located in the left postcentral gyrus (peak voxel at *x* = −56, *y* = −14, *z* = +28), *t*(110) = −4.72, FDR-corrected *p* < 0.001 (see the blue region in Fig. 3a). Moreover, a stronger positive coupling for the OT group, compared to the placebo group, was observed between the left anterior insula and an area (peak voxel at x = +4, y = +64, z = +30) predominantly comprising the right ventromedial prefrontal cortex (vmPFC; 57%) and the right superior frontal gyrus (33%), *t*(110) = 4.37, FDR-corrected *p* < 0.001 (see the red bulb in Fig. 3a). The brain-behavior associations showed significant negative correlations between the insula-postcentral gyrus connectivity and baseline cortisol levels on the next day, *r*(107) = −0.27, *p* = 0.004, and baseline state anxiety levels on the next day, *r*(109) = −0.24, *p* = 0.01. See Fig. 3b, c for scatter plots. Controlling for sex hormones (estradiol, progesterone, and testosterone) using partial correlation did not affect the relationship, all *p*s < 0.007.

### Extinction learning (day 2)

#### Behavior

No significant state anxiety differences were observed between the PP, PO, OP, and OO groups at baseline, *F*(3, 110) = 1.2, *p* = 0.32, or post-MRI, *F*(3, 110) = 0.82, *p* = 0.49. See Supplementary Table 6 for details. Participants were asked post-MRI to rate the intensity and unpleasantness of electric shock pain that they received on day 1 in the scanner. No significant difference was observed between the placebo (4.98 ± 1.00; PP and PO groups pooled) and OT (5.30 ± 1.09; OP and OO groups pooled) groups for the memory of pain intensity, *t*(110) = −1.56, *p* = 0.122, suggesting that the application of intranasal OT prior to fear acquisition (day 1) did not change the recollection of pain intensity after extinction learning on day 2. In contrast, a significant difference was observed between the placebo (5.02 ± 1.76) and OT (5.67 ± 1.67) groups for the memory of pain unpleasantness, *t*(110) = -2.01, *p* = 0.047, indicating that intranasal OT applied on day 1 increased the memory of pain unpleasantness on day 2. Participants were not able to guess better than chance which substance they had received when they were asked on day 1, on day 2, and on day 2 about day 1 (see Supplementary Table 7 for details).

#### Psychophysiology

**Fig. 4:**
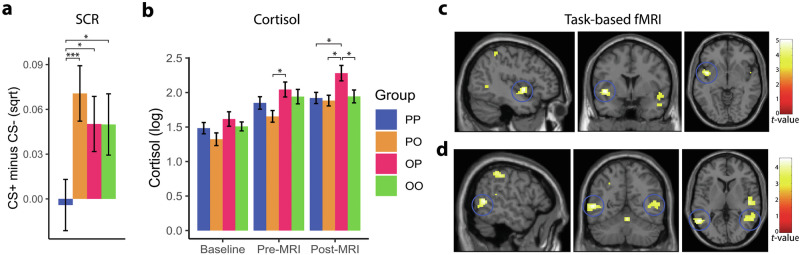
Effects of intranasal oxytocin (OT) on extinction learning. **a** Compared to participants who received placebo on both days (PP), other groups who received OT on day 1 (OP), on day 2 (PO), or on both days (OO) showed a greater skin conductance response (SCR). **b** Salivary cortisol was elevated in the OP group compared to three other groups post-MRI. **c** Whole-brain analysis revealed that the BOLD activation for the CS+ versus CS− contrast was stronger in the left anterior insula of the OP compared to the PP group. **d** Whole-brain analysis showed a stronger differential BOLD signal (CS+ > CS−) in the bilateral middle temporal gyrus for the PO versus PP group. These results suggest that intranasal OT (regardless of being administered prior to fear conditioning or extinction learning) increases anxiety responses towards extinguished conditioned stimulus.

There was a significant between-group difference in SCR, *F*(3, 95) = 3.09, *p* = 0.031. Post-hoc analyses revealed that groups PO (0.07 ± 0.10; *p* = 0.004), OP (0.05 ± 0.09; *p* = 0.047), and OO (0.05 ± 0.10; *p* = 0.043) had significantly higher amplitudes compared to the PP group (−0.01 ± 0.09; see Fig. 4a).

#### fMRI

Whole-brain analysis revealed increased BOLD responses in the OP group, compared to the PP group, towards CS+ versus CS− in the left anterior insula (peak voxel at *x* = −42, *y* = 3, *z* = −4.5), *z* = 4.72, *p*_FWE_ = 0.040, suggesting that the administration of intranasal OT prior to fear conditioning (day 1) led to a hypermetabolism of the anterior insula during extinction learning (day 2). See Fig. 4c for anatomical localization. No other cluster passed the significance threshold. See Supplementary Table 8 for statistical and stereotaxic details. For the PO compared to the PP group for the CS+ versus CS− contrast, increased BOLD activations were observed in the bilateral middle temporal gyrus. See Fig. 4d for localization and Supplementary Table 9 for statistical and stereotaxic details. For the group that received intranasal OT on both days (group OO) compared to the PP group towards CS+ versus CS−, no significant difference was observed for the contrast of CS+ versus CS−. See Supplementary Table 10 for details.

#### Hormones

At baseline, no significant differences in cortisol levels were observed across groups, *F*(3, 108) = 1.94, *p* = 0.13 (Fig. 4b). At pre-MRI, a significant between-group difference emerged, *F*(3, 108) = 2.97, *p* = 0.035. Post-hoc analysis showed significantly higher cortisol levels for the OP (2.04 ± 0.58) compared to the PO group (1.65 ± 0.46), FDR-corrected *p* = 0.025. At post-MRI, the cortisol levels in OP participants (2.28 ± 0.60) were higher compared to the other groups: PP (1.92 ± 0.43; FDR-corrected *p* = 0.025), PO (1.88 ± 0.42; FDR-corrected *p* = 0.025), and OO (1.94 ± 0.47; FDR-corrected *p* = 0.035).

## Discussion

This randomized, double-blind, placebo-controlled, two-day fMRI study demonstrated that intranasal OT administered to females had impairing effects on extinction learning, regardless of whether it was administered prior to fear conditioning or extinction learning. The administration of intranasal OT prior to fear conditioning did not affect fear conditioning, but (a) immediately after fear conditioning, resting-state functional connectivity was modulated. Specifically, we found that the OT group, compared to the placebo group, showed a stronger positive connectivity between the left anterior insula and the right superior frontal gyrus and vmPFC as well as a stronger negative connectivity between the left anterior insula and the left postcentral gyrus. The insula-postcentral gyrus connectivity showed a negative correlation with anxiety-related measures (self-reported anxiety and salivary cortisol) on the next day (extinction learning); (b) The next day, during extinction learning, we found that the BOLD activation in the anterior insula was increased, accompanied by heightened SCRs and followed by increased cortisol levels post-MRI. Administration of intranasal OT prior to extinction learning increased the BOLD activation in the bilateral middle temporal gyrus accompanied by increased SCRs.

Similar to rodent findings [12] when OT was infused into the ventricles of the brain, we found no significant effects of intranasal OT on fear conditioning. No behavioral (self-reported anxiety), physiological (SCR), neuronal (BOLD; CS+ > CS−), or hormonal (salivary cortisol) differences were observed between the OT and placebo groups (Fig. 2). It should be noted that in addition to the general and wide-range effects of OT via the cerebrospinal fluid [9, 13], OT can exert region-specific effects via axonal projections. Infusion of OT into the central or basolateral nuclei of the amygdala prior to conditioning impaired fear acquisition and resulted in reduced freezing the following day [23]. Moreover, optogenetic stimulation of hypothalamic OT neurons prior to extinction learning resulted in the release of endogenous OT in the rat central amygdala and resulted in the reduction of fear expression [24–26]. These findings suggest that the modulatory effects of OT on the brain may vary from widespread to region-specific targets depending on how OT is endogenously released or exogenously administered.

In contrast to rodent findings in which OT administration prior to fear conditioning facilitated extinction learning the following day [12], we found that intranasal OT, in fact, reduced extinction learning. Participants who received intranasal OT, as compared to those who received placebo, prior to fear conditioning (day 1) demonstrated heightened SCRs and hyperactivation of the left anterior insula on the next day during extinction learning (Fig. 4c). The anterior insula has consistently been reported in human fear extinction fMRI studies [5], with higher activation linked to stronger fear expression. It is thought that the posterior part of the insula is more involved in the primary detection of interoceptive signals and the anterior part is involved in conscious interoceptive awareness as a result of the integration of emotional and cognitive signals collected from various regions such as amygdala, anterior cingulate cortex, and striatum [27–29]. The OT system has been suggested to play a modulatory role in sensory processing including processing in the insula [30, 31]. As an exploratory analysis, we performed multiple regression to evaluate the relationship between SCR (CS+ minus CS−) and differential BOLD activation (CS+ > CS−) for all participants during extinction learning on day 2. We found a significant positive correlation between SCR and bilateral insula activation, confirming that stronger insular activity is associated with higher SCR (see Supplementary Table 11 for details).

One explanation for reduced extinction learning—as opposed to facilitated extinction learning in rodents when OT was infused intracerebroventricularly—when intranasal OT was administered prior to conditioning may be due to the complex interaction of intranasal OT with the central and peripheral OT systems. There is strong evidence that intranasal OT reaches the general blood circulation and it might directly reach the brain at physiologically significant amounts [19]. A recent study showed that the use of a vasoconstrictor to prevent intranasal OT from entering the general circulation significantly reduced OT effects on brain activity [20], suggesting that the intranasal OT effects on the brain might be primarily exerted via peripheral mechanisms [32]. However, these mechanisms remain largely unknown and uninvestigated. One hypothesis to explain these observations is related to a possible feedback mechanism that regulates the concentration levels of endogenous OT in the central nervous system and in the periphery [9]. According to this hypothesis, elevated levels of OT in the periphery may trigger a negative feedback loop leading to reduced secretion of OT in the brain and consequently lowering OT concentrations in the brain and increasing the risk of impaired fear extinction.

To investigate the effects of intranasal OT (administered prior to fear conditioning) on fear memory consolidation, we measured resting-state functional connectivity immediately after fear conditioning on day 1 [33]. We chose the left anterior insula as the seed region as significant differences were observed between the OP (receiving OT on day 1 and placebo on day 2) and the PP (receiving placebo on both days) groups during extinction learning (Fig. 4c). Long-term memory formation requires time and the involvement of various networks [15, 34]. We observed a stronger negative correlation between the insula and the postcentral gyrus in the OT as compared with the placebo group (Fig. 3). The postcentral gyrus receives somatic sensations from the contralateral side of the body. Since the electrodes for delivering electric stimulation were placed on the right forearm, this might explain the involvement of the left (but not the right) postcentral gyrus. The laterality effect can be tested in future studies by placing the electrodes on left and right body parts in different participants. Moreover, significant correlations were observed between the insula-postcentral gyrus connectivity and anxiety levels (salivary cortisol and self-reported anxiety) on the next day (extinction learning), suggesting a potential link between postconditioning oxytocinergic modulation of fear memory consolidation.

Similar to animal findings [12], intranasal OT demonstrated anxiogenic effects reflected in SCR and brain activities when administered prior to extinction learning, suggesting that OT impairs fear extinction. This observation is also in line with the findings of a meta-analysis showing the anxiogenic effects of intranasal OT in victims of recent traumatic experiences [10]. The observed increased activity over the bilateral middle temporal gyrus is consistent with the findings of a meta-analysis showing that intranasal OT modulates the processing of threatening stimuli within the temporal lobes [35].

Our study has several limitations. The sample was only comprised of healthy females and due to the sex-specific effects of OT [36–38], the findings cannot be generalized to males. We used only one dose of intranasal OT (24 IU)—which is the most common dosage form [39, 40]—and therefore no dose-response relationship can be determined from the data. Although analyzing the extinction learning as one single block can improve statistical power and provide a broad overview of neural activity associated with the extinction process, dividing extinction learning into early and late phases can better examine the temporal dynamics of extinction learning. The current study is lacking an extinction recall phase which is necessary to evaluate the retention of fear extinction memories. (Currently used synthetic OT analogs in humans such as Syntocinon and Pitocin have poor penetrability across the blood-brain barrier [32]). To improve OT delivery to the brain and to separate the central and peripheral effects, new synthetic OT analogs with high penetrability may be beneficial [41]. In contrast to anxiolytic effects of OT in rodents [12], we observed anxiogenic effects of intranasal OT on extinction learning when administered prior to conditioning. Future studies could use OT antagonists (e.g., atosiban) to test whether the direction of the effect is reversed, as in rodent findings [12], resulting in the facilitation of extinction learning. Different conditioning and extinction paradigms capture distinct behavioral and neural mechanisms by engaging various aspects of learning, memory, and neural circuitry. Utilizing a range of paradigms can be beneficial, as it provides a more comprehensive understanding of these processes, highlights specific contributions of different neural pathways, and allows for more robust and generalizable findings.

In conclusion, we provide initial evidence that intranasal OT application may impair extinction learning, regardless of the timepoint of application. Importantly, intranasal OT did not affect fear conditioning, but modulated insular functional connectivity immediately after conditioning and impaired extinction learning on the next day. The anxiogenic (rather than the anxiolytic) effects of OT observed in rodents might be due to the interaction between the peripheral and central OT signaling [9]. Similar to rodent findings, OT administration prior to extinction learning (day 2) was anxiogenic and reduced extinction learning. These results demonstrate the modulatory effects of OT on fear extinction memory. On this stage of research OT cannot be recommended as a pharmacological agent or adjunct for exposure therapy in patients with anxiety and stress-related disorders.

## Supplementary information

Supplementary Materials

## Data Availability

The data of this study are available on reasonable request from the corresponding author. The data are not publicly available due to privacy or ethical restrictions.
