# Supplementary material for: Effects of intranasal oxytocin on fear extinction learning
Source: Neuropsychopharmacology. 2024 Sep 24;50(3):548–55. doi: 10.1038/s41386-024-01996-y (PMC11735929; doi:10.1038/s41386-024-01996-y)
Supplement: Supplementary file 1 — Supplementary Materials [file 41386_2024_1996_MOESM1_ESM.docx]

# Supplementary Materials

**Effects of intranasal oxytocin on fear extinction learning**

Mahmoud Rashidi^1,2^, Joe J. Simon^3^, Katja Bertsch^4^, Gerhard Vincent Wegen^1^, Beate Ditzen^5^, Herta Flor^6^, Valery Grinevich^7,8^, Robert Christian Wolf^1*^, Sabine C. Herpertz^1,9*^

^1^ Department of General Psychiatry, Center for Psychosocial Medicine, Heidelberg University, Heidelberg, Germany; ^2^ Department of Psychiatry and Behavioral Health, Wexner Medical Center, The Ohio State University, Ohio, USA; ^3^ Department of General Internal Medicine and Psychosomatics, Center for Psychosocial Medicine, Heidelberg University, Heidelberg, Germany; ^4^ Department of Psychology, Julius Maximilians University of Wuerzburg, Wuerzburg, Germany; ^5^ Institute of Medical Psychology, Center for Psychosocial Medicine, Heidelberg University, Heidelberg, Germany; ^6^ Institute of Cognitive and Clinical Neuroscience, Central Institute of Mental Health, Medical Faculty Mannheim, Heidelberg University, Mannheim, Germany; ^7^ Department of Neuropeptide Research in Psychiatry, Central Institute of Mental Health, Medical Faculty Mannheim, Heidelberg University, Mannheim, Germany; ^8^ Center for Neuroinflammation and Cardiometabolic Diseases, Georgia State University, Atlanta, USA; DZPG, German Center for Mental Health

^*^ These authors equally contributed to this work.

**Corresponding author:** Mahmoud Rashidi, Ph.D.

Heidelberg University, Department of General Psychiatry

Vossstr. 4, 69115 Heidelberg, Germany

Tel: +49 6221 56-34881; Fax: +49 6221 56-5998;

Email: [rashidi@uni-heidelberg.de](mailto:rashidi@uni-heidelberg.de)

## Data acquisition and preprocessing

*Behavior.* Participants reported their current anxiety levels using the State-Trait Anxiety Inventory (STAI)(1) separately for baseline and post-MRI. For measuring the memory of pain intensity and unpleasantness, these questions were asked on Day 2 post-MRI: “How high was the pain intensity/unpleasantness of the electric stimulus yesterday in the MRI on a scale of 0-10? (Assume the pain intensity/unpleasantness during pain threshold determination was at 5.”

*Psychophysiology.* Skin conductance responses (SCRs) were recorded using an MR-compatible 0.5-volt constant voltage sensor (BrainProducts, Gilching, Germany) with Ag/AgCl electrodes and filled with isotonic paste. They were placed on the distal phalanges of the second and third fingers of participants’ left hand. Responses were amplified and digitized at 5000 Hz using BrainAmp ExG MR (BrainProducts, Gilching, Germany). Preprocessing included median filtering to remove spike artifacts from the scanner, segmentation, baseline correction using the interval 2 s prior to CS onset, signed square root transformation, and peak detection from 0-5 s relative to CS onset. Four participants were identified as non-responders and were excluded as they showed no conductance level above 0.02 μS in any trial (CS- or CS+). Differential SCRs were calculated by subtracting the mean of CS- trials from the mean of CS+ trials for each participant. For fear conditioning, all 16 trials for each CS type were included. For extinction learning, the first two CS- and CS+ trials were excluded as they are more reflective of fear expression (2).

*Task-based fMRI.* Structural and functional images were acquired using a 3-Tesla scanner (MAGNETOM Prisma, Siemens, Erlangen, Germany) with a 64-channel head coil. Forty-six slices (voxel size = 3 × 3 × 2.5 mm, 1 mm gap) were acquired in each volume using a T2-weighted echo-planar imaging sequence (repetition time = 2560 ms, echo time = 30 ms, field of view = 192 x 192mm, flip angle = 90°, GRAPPA acceleration factor = 2). Preprocessing and analyses were conducted using SPM12 (3) version 7771. Preprocessing included slice timing correction, realignment, co-registration to individual anatomical images, segmentation, MNI-space normalization, and smoothing using a 6-mm full-width at half maximum Gaussian kernel.

*Resting-state fMRI.* Data acquisition parameters were identical to the task-based fMRI. A total of 116 volumes were recorded, resulting in ~5 min of data. Participants were instructed to remain as motionless as possible, to keep their eyes closed, to not think of anything in particular, and to not fall asleep. Preprocessing and analyses were carried out using SPM12 and CONN toolbox (4) release 21.a. See Supplementary Materials for a detailed description of the procedure. Briefly, functional and anatomical data were preprocessed using a flexible preprocessing pipeline (5) including realignment with correction of susceptibility distortion interactions, slice timing correction, outlier detection, direct segmentation, MNI-space normalization, smoothing, and denoising.

*Hormones.* For measuring endogenous OT, estradiol, progesterone, and testosterone, a blood sample was collected into a BD Vacutainer tube (K2E EDTA, 10 ml). Blood plasma was obtained by centrifugation (1500 g, 15 min, +4 °C) and aliquoted samples were stored immediately at -80°C. For measuring cortisol, participants provided at least 1 mL saliva via passive drool through a straw into SaliCaps (IBL International, Hamburg, Germany). The saliva samples were stored immediately at -80 °C. Samples were analyzed using enzyme-linked immunosorbent assay (ELISA) according to the manufacturer’s protocol at the biochemical lab in the Institute of Medical Psychology at Heidelberg University (for details, see Supplementary Table 1).

# Pain threshold determination

The pain threshold for each participant was determined individually. A bipolar bar electrode (E.SB010/4 mm, Digitimer, Hertfordshire, UK) was placed on the right forearm. The electric current of an electric stimulus with a duration of 2 ms and voltage of 400 volts generated by a constant current stimulator (Digitimer DS7, UK) was gradually increased until participants reported that the stimulus was extremely unpleasant but not painful. Afterwards, participants were told to assume that this level is of a pain intensity of 0 and the electric stimulus intensity will be increased gradually until its intensity will be perceived as 5 where 0 is not painful at all and 10 is the worst pain imaginable. Participants were told that in case a stimulus has reached an intensity above 5, they can request to lower the stimulus intensity. For 0 to 10 mA, 12 to 40 mA, and 45 to 100 mA, the current was increased in 1-, 2-, and 5-mA steps. The place of the bar electrode was marked by a pen and later an MR-compatible bar electrode with similar physical characteristics was placed on the forearm inside the MRI scanner.

# Resting-state fMRI functional connectivity

The description of preprocessing and statistical analysis is generated by CONN toolbox and modified by the authors of this study.

*Preprocessing.* Functional data were realigned using SPM realign & unwarp procedure (6), where all scans were co-registered to a reference image (first scan of the first session) using a least squares approach and a 6-parameter (rigid body) transformation (7), and resampled using b-spline interpolation to correct for motion and magnetic susceptibility interactions. Temporal misalignment between different slices of the functional data (acquired in descending order) was corrected following SPM slice-timing correction procedure (8, 9), using sinc temporal interpolation to resample each slice BOLD timeseries to a common mid-acquisition time. Potential outlier scans were identified using ART (10) as acquisitions with framewise displacement above 0.9 mm or global BOLD signal changes above 5 standard deviations (11), and a reference BOLD image was computed for each subject by averaging all scans excluding outliers. Functional and anatomical data were normalized into standard MNI space, segmented into grey matter, white matter, and CSF tissue classes, and resampled to 2 mm isotropic voxels following a direct normalization procedure (12) using SPM unified segmentation and normalization algorithm (13, 14) with the default IXI-549 tissue probability map template. Last, functional data were smoothed using spatial convolution with a Gaussian kernel of 8 mm full width at half maximum (FWHM).

*Denoising.* In addition, functional data were denoised using a standard denoising pipeline (5) including the regression of potential confounding effects characterized by white matter timeseries (5 CompCor noise components), CSF timeseries (5 CompCor noise components), motion parameters and their first order derivatives (12 factors) (15), outlier scans (below 30 factors) (11), session effects and their first order derivatives (2 factors), and linear trends (2 factors) within each functional run, followed by bandpass frequency filtering of the BOLD timeseries (16) between 0.008 Hz and 0.09 Hz. CompCor (17, 18) noise components within white matter and CSF were estimated by computing the average BOLD signal as well as the largest principal components orthogonal to the BOLD average, motion parameters, and outlier scans within each subject's eroded segmentation masks. From the number of noise terms included in this denoising strategy, the effective degrees of freedom of the BOLD signal after denoising were estimated to range from 25.2-37.8 (average 37.3) across all subjects.

The strength of functional connectivity was represented by Fisher-transformed bivariate correlation coefficients from a weighted general linear model. To compensate for possible transient magnetization effects at the beginning of each run, individual scans were weighted by a step function convolved with an SPM canonical hemodynamic response function and rectified. In the second-level analysis, for each individual voxel a separate general linear model was estimated, with first-level connectivity measures at this voxel as dependent variables, and group (placebo or OT) as independent variable. Voxel-level hypotheses were evaluated using multivariate parametric statistics with random-effects across participants and sample covariance estimation across multiple measurements. Inferences were performed at the level of individual clusters (groups of contiguous voxels). Cluster-level inferences were based on parametric statistics from Gaussian Random Field theory (19). Results were thresholded using a combination of a cluster-forming *p* < 0.001 voxel-level threshold, and a family-wise corrected *p* < 0.05 cluster-size threshold (20).


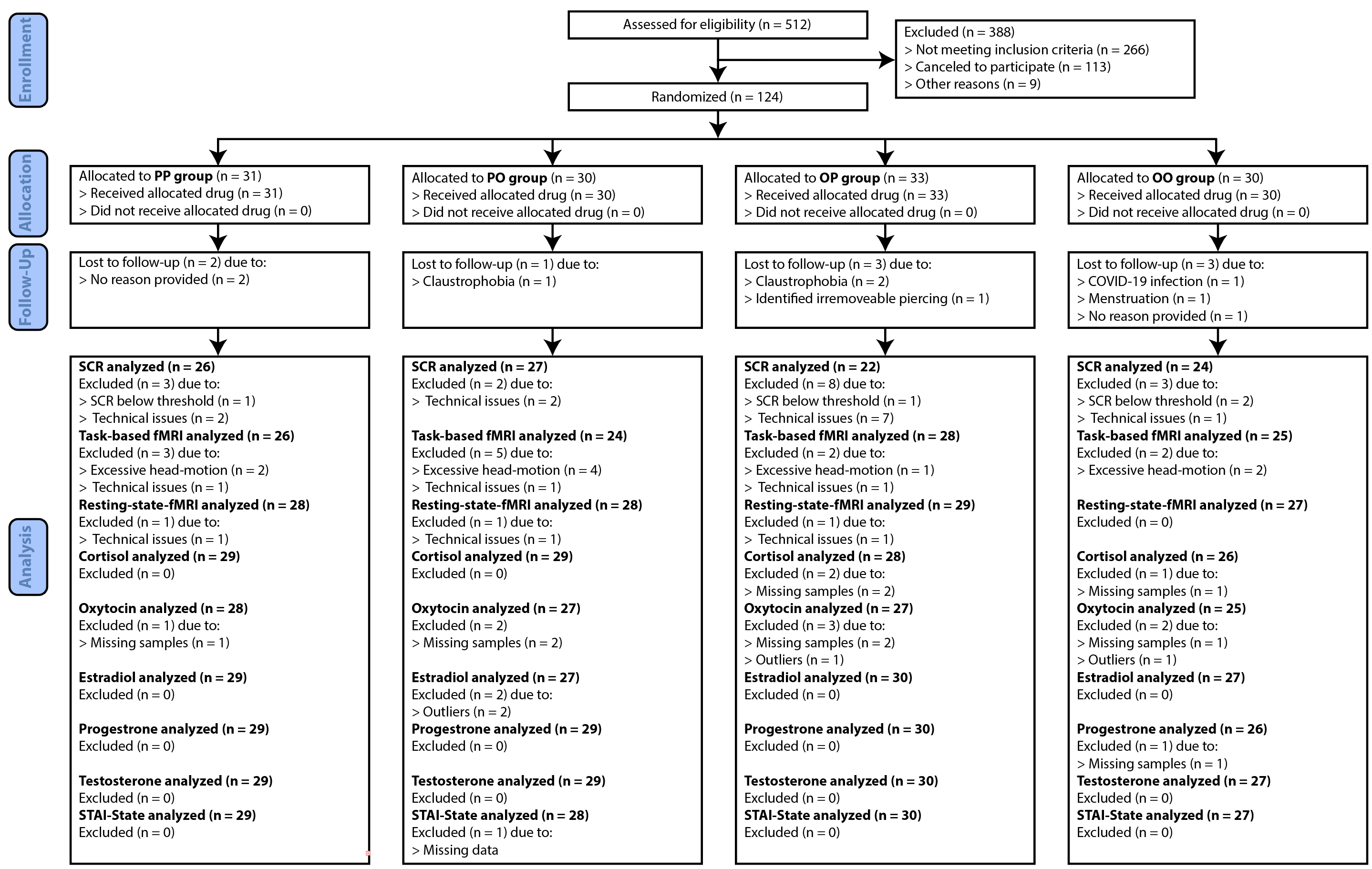


Supplementary Figure 1. The CONSORT flow chart. On Day 1 (fear conditioning), participants received either intranasal placebo (PP and PO groups) or oxytocin (OP and OO groups), resulting in two groups (placebo or oxytocin groups). On Day 2 (extinction learning), participants received either intranasal placebo (PP and OP groups) or oxytocin (PO and OO groups), resulting in four groups given different substances administered on Day 1. SCR: skin conductance response; STAI: the State-Trait Anxiety Inventory.


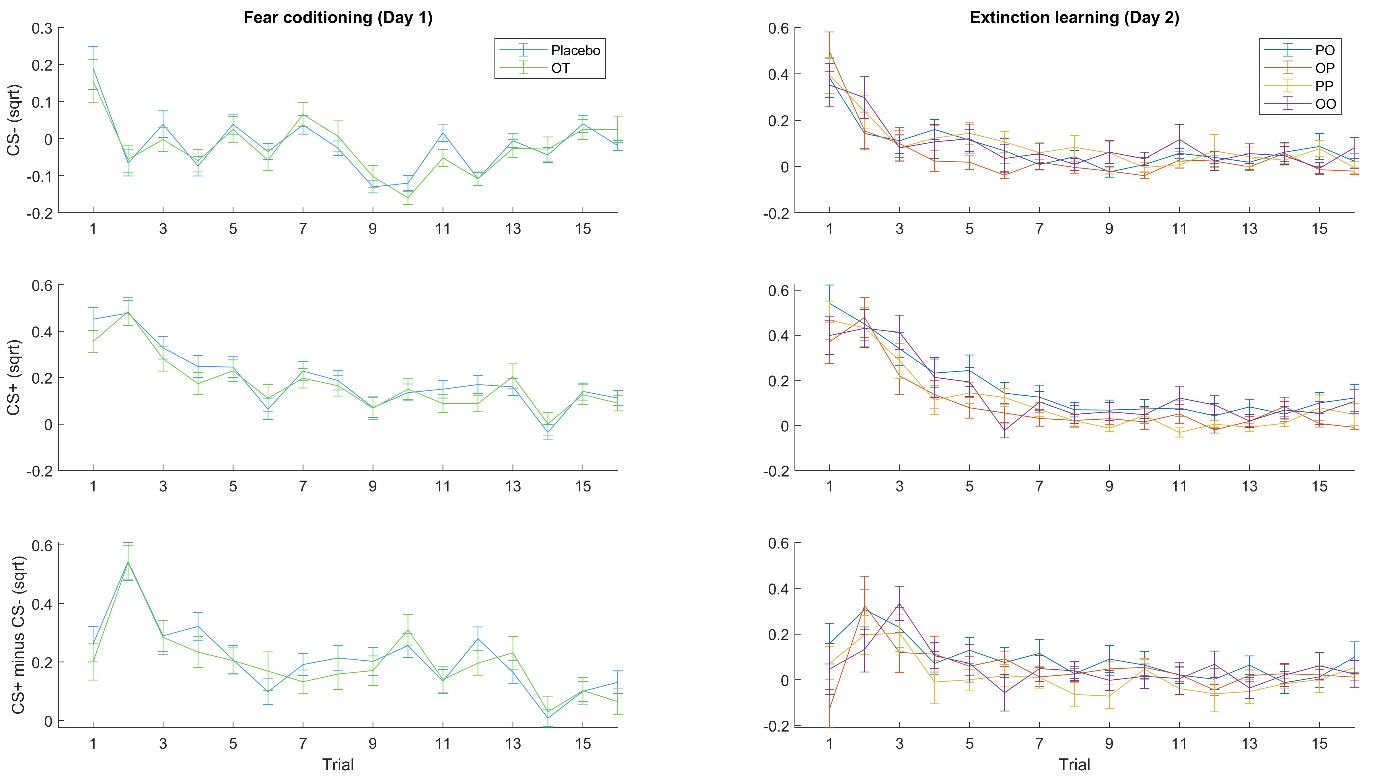


Supplementary Figure 2. Skin conductance response to conditioned stimulus (CS) unpaired with electric stimuli (CS-), paired with electric stimuli (CS+), and subtraction of CS- from CS+.


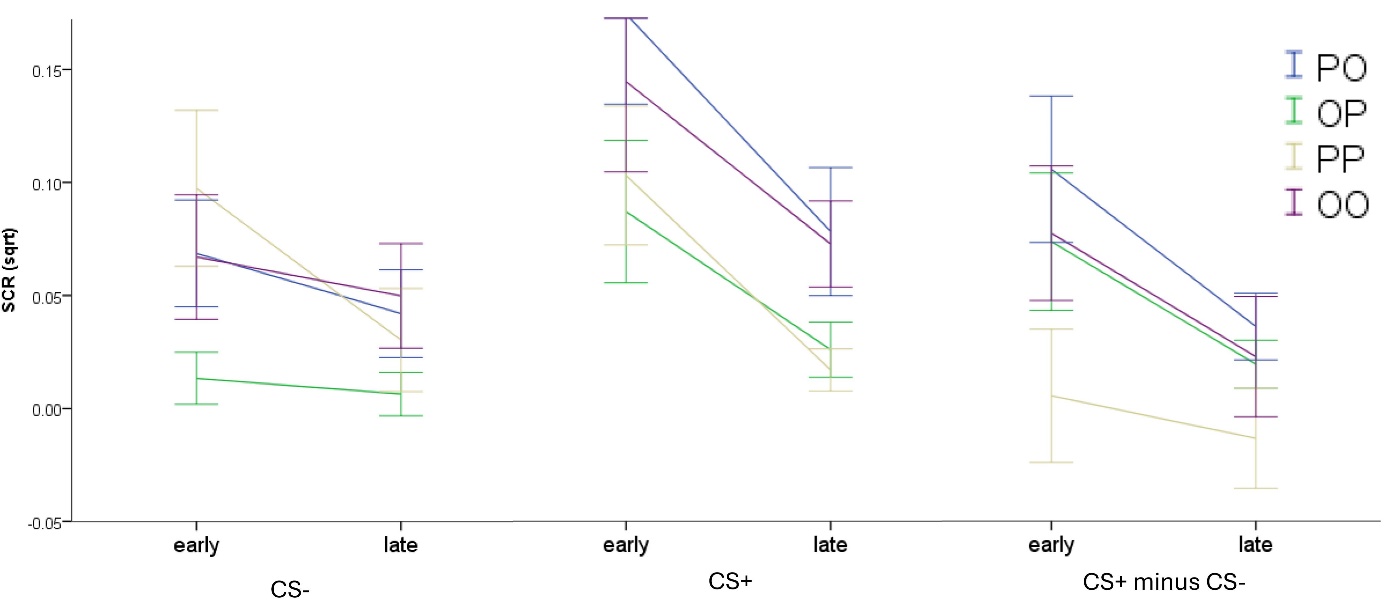


Supplementary Figure 3. Skin conductance response (SCR) of the early versus late phase of extinction learning. A 2 × 2 × 4 repeated-measures ANOVA was conducted with the within-subject factors of stimulus type (CS- or CS+) and phase (early or late), and the between-subject factor of treatment (PO, OP, PP, and OO). The main effects of phase, *F*(1, 96) = 22.64, *p* < 0.001, and stimulus type, *F*(1, 96) = 19.03, *p* < 0.001, were significant. The main effect of treatment was not significant, *F*(3, 96) = 1.65, *p* = 0.18. The interaction between phase and treatment was not significant, *F*(3, 96) = 0.67, *p* = 0.57. The interaction between stimulus type and phase, *F*(1, 96) = 7.69, *p* = 0.007, and stimulus type and treatment, *F*(3, 96) = 3.05, *p* = 0.032 were significant. The interaction between stimulus type, phase, and treatment was not significant, *F*(3, 96) = 0.39, *p* = 0.76.


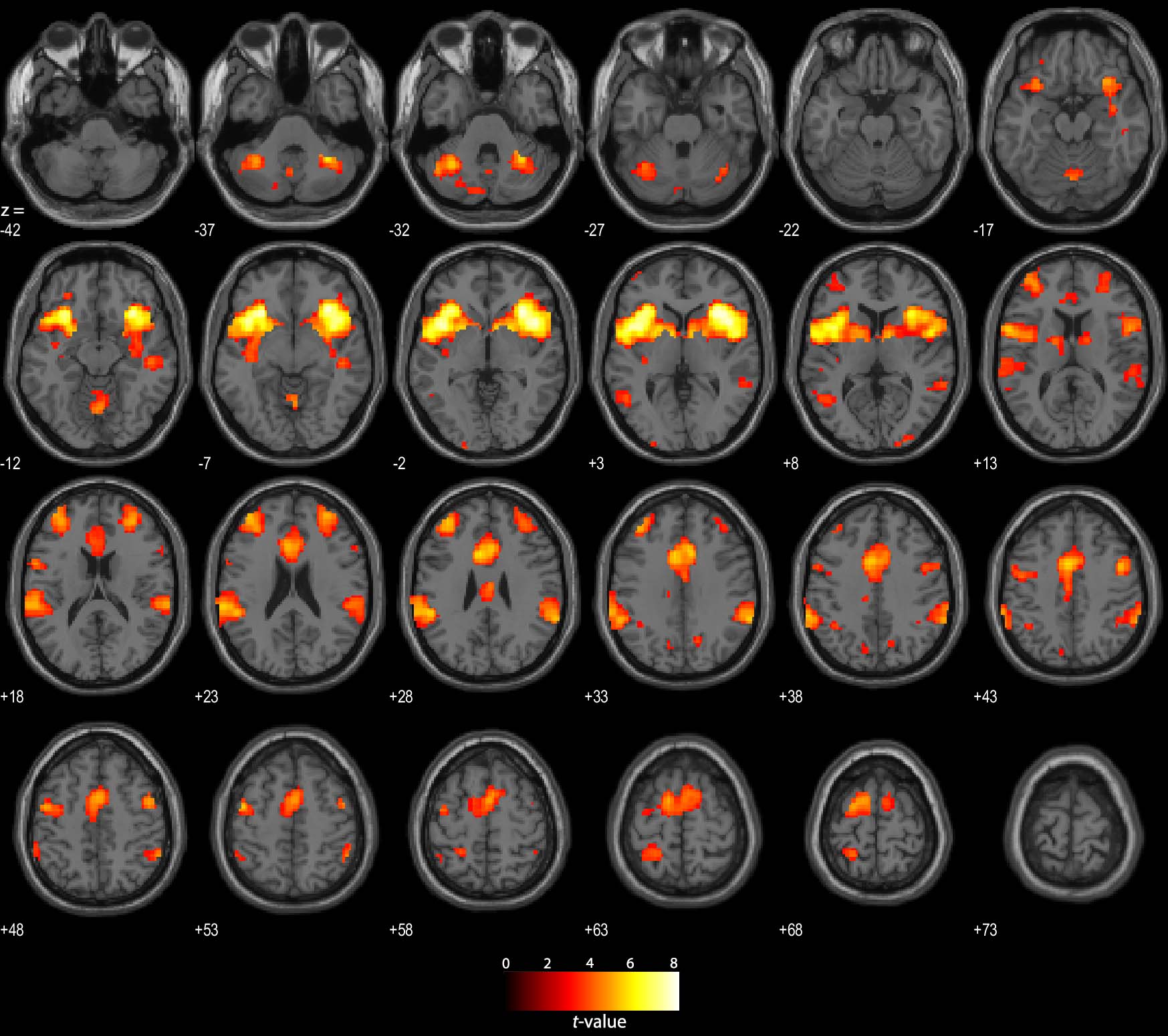


Supplementary Figure 4. Differential BOLD responses (CS+ > CS-) during fear conditioning for all participants (placebo and oxytocin groups combined). *T*-maps were thresholded at *p* < 0.001 (voxel-level, uncorrected), with a minimum cluster size of 10 adjacent voxels. Regions were considered significant at the cluster-level for *p* < 0.05, FWE-corrected.

Supplementary Table 1. Assessment of hormones. CV: coefficient of variation.

| Hormone | Kit model | Kit manufacturer, country | Intra-assay  CV (%) | Inter-assay  CV (%) | Duplicates  (%) |
| --- | --- | --- | --- | --- | --- |
| Oxytocin | ADI-901-153A | Enzo Life Sciences, NY, USA | 6.23 | 11.83 | 48 |
| Estradiol | RE52041 | IBL International, Hamburg, Germany | 4.95 | 6.08 | 44 |
| Progesterone | RE52231 | IBL International, Hamburg, Germany | 3.77 | 11.66 | 44 |
| Testosterone | RE52151 | IBL International, Hamburg, Germany | 4.71 | 2.1 | 44 |
| Cortisol | DES6611 | Demeditec Diagnostics, Kiel, Germany | 2.82 | 5.17 | 11 |

Supplementary Table 2. Baseline concentration of hormones on Day 1 in each group.

|  | Group | | d.f. | t | p |
| --- | --- | --- | --- | --- | --- |
|  | Placebo  mean ± SD | Oxytocin  mean ± SD |  |  |  |
| Oxytocin (pg/mL) | 61.37 ± 33.07 | 64.17 ± 34.97 | 105 | -0.43 | 0.67 |
| Estradiol (pg/mL) | 125.15 ± 77.23 | 116.13 ± 67.29 | 111 | 0.66 | 0.51 |
| Progesterone (ng/mL) | 5.96 ± 5.70 | 6.76 ± 7.11 | 112 | -0.66 | 0.51 |
| Testosterone (ng/mL) | 0.45 ± 0.14 | 0.43 ± 0.12 | 113 | 0.85 | 0.40 |
| Cortisol (ng/mL) | 1.57 ± 0.47 | 1.70 ± 0.41 | 110 | -1.52 | 0.13 |

Supplementary Table 3. Baseline hormonal concentrations on Day 1 and Day 2.

|  |  |  | Day 1  mean ± SD | Day 2  mean ± SD | *d.f.* | *t* | *p* |
| --- | --- | --- | --- | --- | --- | --- | --- |
| Group | | |  |  |  |  |  |
|  | **Placebo** | |  |  |  |  |  |
|  |  | Oxytocin (pg/mL) | 61.37 ± 33.07 | 58.57 ± 22.21 | 54 | 0.58 | 0.57 |
|  |  | Estradiol (pg/mL) | 125.15 ± 77.23 | 118.93 ± 72.15 | 55 | 1.06 | 0.29 |
|  |  | Progesterone (ng/mL) | 5.96 ± 5.70 | 6.61 ± 6.95 | 57 | -1.31 | 0.19 |
|  |  | Testosterone (ng/mL) | 0.45 ± 0.14 | 0.45 ± 0.14 | 57 | 0.43 | 0.67 |
|  |  | Cortisol (ng/mL) | 1.57 ± 0.47 | 1.40 ± 0.47 | 56 | 3.67 | <0.001 |
|  | **Oxytocin** | |  |  |  |  |  |
|  |  | Oxytocin (pg/mL) | 64.17 ± 34.97 | 60.98 ± 25.67 | 51 | 0.69 | 0.49 |
|  |  | Estradiol (pg/mL) | 116.13 ± 67.29 | 114.05 ± 61.47 | 56 | 0.37 | 0.71 |
|  |  | Progesterone (ng/mL) | 6.76 ± 7.11 | 6.67 ± 6.20 | 55 | 0.18 | 0.86 |
|  |  | Testosterone (ng/mL) | 0.43 ± 0.12 | 0.42 ± 0.11 | 56 | 1.47 | 0.15 |
|  |  | Cortisol (ng/mL) | 1.70 ± 0.41 | 1.57 ± 0.47 | 54 | 3.03 | 0.004 |

Supplementary Table 4. Statistical and stereotaxic coordinates of clusters and voxels for the differential contrast of “CS+ > CS-“ during fear conditioning for all participants (placebo and oxytocin groups combined).

| Cluster-level | |  | Voxel-level | | |  | Peak voxel MNI | | |  | Region |
| --- | --- | --- | --- | --- | --- | --- | --- | --- | --- | --- | --- |
| Voxel count | *p*_FWE_ |  | *t* | *z*_max_ | *p_FWE_* |  | x | y | z |  |  |
| 3227 | <0.001 |  | 8.484 | 7.354 | <0.001 |  | 45 | 12 | 0.5 |  | Right insula |
|  |  |  | 8.461 | 7.339 | <0.001 |  | 33 | 21 | 0.5 |  |  |
|  |  |  | 8.218 | 7.174 | <0.001 |  | 33 | 24 | -9.5 |  |  |
| 150 | 0.009 |  | 6.670 | 6.057 | <0.001 |  | 33 | -51 | -34.5 |  | Right cerebellum crus 1 |
|  |  |  | 4.367 | 4.169 | 0.188 |  | 39 | -60 | -29.5 |  |  |
|  |  |  | 4.059 | 3.897 | 0.418 |  | 33 | -69 | -27 |  |  |
| 1675 | <0.001 |  | 6.178 | 5.676 | <0.001 |  | -6 | 15 | 30.5 |  | Left anterior cingulate cortex |
|  |  |  | 6.018 | 5.550 | <0.001 |  | -3 | 6 | 43 |  |  |
|  |  |  | 5.925 | 5.476 | <0.001 |  | 6 | 18 | 30.5 |  |  |
| 850 | <0.001 |  | 6.071 | 5.592 | <0.001 |  | -51 | -39 | 25.5 |  | Left supramarginal gyrus |
|  |  |  | 5.748 | 5.334 | 0.001 |  | -63 | -42 | 38 |  |  |
|  |  |  | 5.722 | 5.313 | 0.002 |  | -66 | -33 | 23 |  |  |
| 574 | <0.001 |  | 6.004 | 5.539 | <0.001 |  | 63 | -45 | 30.5 |  | Right supramarginal gyrus |
|  |  |  | 5.465 | 5.103 | 0.004 |  | 60 | -48 | 40.5 |  |  |
|  |  |  | 5.232 | 4.910 | 0.010 |  | 66 | -30 | 18 |  |  |
| 253 | <0.001 |  | 5.725 | 5.315 | 0.002 |  | -33 | -54 | -34.5 |  | Left cerebellum crus 1 |
|  |  |  | 5.323 | 4.985 | 0.007 |  | -48 | -60 | -34.5 |  |  |
|  |  |  | 3.745 | 3.615 | 0.738 |  | -9 | -81 | -32 |  |  |
| 392 | <0.001 |  | 5.710 | 5.303 | 0.002 |  | -36 | 45 | 28 |  | Left middle frontal gyrus |
|  |  |  | 5.387 | 5.039 | 0.006 |  | -36 | 54 | 15.5 |  |  |
|  |  |  | 3.518 | 3.408 | 0.915 |  | -39 | 60 | 3 |  |  |
| 127 | 0.018 |  | 5.414 | 5.061 | 0.005 |  | 45 | 3 | 43 |  | Right precentral gyrus |
|  |  |  | 5.268 | 4.940 | 0.009 |  | 48 | 0 | 50.5 |  |  |
| 301 | <0.001 |  | 5.410 | 5.057 | 0.005 |  | 33 | 48 | 20.5 |  |  |
|  |  |  | 4.589 | 4.363 | 0.095 |  | 36 | 42 | 28 |  |  |
|  |  |  | 3.649 | 3.528 | 0.825 |  | 39 | 39 | 35.5 |  |  |
| 115 | 0.025 |  | 5.163 | 4.852 | 0.013 |  | 0 | -69 | -14.5 |  | Vermis XI |
| 13 | 0.801 |  | 4.414 | 4.211 | 0.163 |  | 0 | -63 | -34.5 |  | Vermis XIII |
| 19 | 0.674 |  | 4.163 | 3.990 | 0.326 |  | 15 | -66 | 35.5 |  | Right cuneus |
| 15 | 0.759 |  | 4.120 | 3.951 | 0.363 |  | 24 | -96 | 10.5 |  | Right superior occipital gyrus |
| 32 | 0.435 |  | 4.067 | 3.905 | 0.410 |  | -36 | -51 | 35.5 |  | Left Angular gyrus |
| 10 | 0.861 |  | 3.981 | 3.827 | 0.495 |  | -21 | -102 | -2 |  | Left middle occipital gyrus |
|  |  |  | 3.603 | 3.486 | 0.860 |  | -27 | -99 | 3 |  | Left middle occipital gyrus |
| 12 | 0.821 |  | 3.855 | 3.714 | 0.626 |  | -36 | -21 | 5.5 |  | Left Heschl’s gyrus |
| 19 | 0.674 |  | 3.699 | 3.574 | 0.781 |  | -9 | -72 | 35.5 |  | Left cuneus |
|  |  |  | 3.445 | 3.342 | 0.948 |  | -9 | -78 | 43 |  | Left superior occipital gyrus |

Supplementary Table 5. No significant difference between placebo and oxytocin groups were observed for the contrast CS+ > CS-.

| Cluster-level | |  | Voxel-level | | |  | Peak voxel MNI | | |  | Region |
| --- | --- | --- | --- | --- | --- | --- | --- | --- | --- | --- | --- |
| Voxel count | *p*_FWE_ |  | *t* | *z*_max_ | *p*_FWE_ |  | x | y | Z |  |  |
| 53 | 0.203 |  | 4.784 | 4.531 | 0.050 |  | -63 | -57 | 28 |  | Left supramarginal gyrus |
|  |  |  | 3.407 | 3.307 | 0.962 |  | -63 | -45 | 35.5 |  |  |
| 79 | 0.081 |  | 4.714 | 4.471 | 0.063 |  | -48 | 30 | 30.5 |  | Left inferior frontal gyrus |
|  |  |  | 4.362 | 4.165 | 0.190 |  | -54 | 24 | 25.5 |  |  |
| 13 | 0.801 |  | 3.833 | 3.695 | 0.649 |  | -45 | 27 | -2 |  | Left inferior frontal gyrus |
| 5 | 0.946 |  | 3.533 | 3.422 | 0.906 |  | -57 | 27 | 13 |  | Left inferior frontal gyrus |
| 2 | 0.981 |  | 3.359 | 3.262 | 0.975 |  | -51 | -48 | 28 |  | Left supramarginal gyrus |
| 4 | 0.960 |  | 3.343 | 3.248 | 0.978 |  | -18 | 57 | 23 |  | Left superior frontal gyrus |
| 2 | 0.981 |  | 3.241 | 3.154 | 0.992 |  | -51 | -42 | 45.5 |  | Left inferior parietal lobule |
| 1 | 0.989 |  | 3.202 | 3.117 | 0.995 |  | -51 | 9 | 10.5 |  | Left inferior opercular gyrus |

Supplementary Table 6. STAI state anxiety on Day 2.

|  | | n | Mean | SD | SEM | Confidence Interval (95%) | |
| --- | --- | --- | --- | --- | --- | --- | --- |
|  |  |  |  |  |  | Lower Bound | Upper Bound |
| **Baseline** | |  |  |  |  |  |  |
|  | PP | 29 | 32.207 | 4.647 | 0.863 | 30.439 | 33.975 |
|  | PO | 28 | 32.214 | 9.343 | 1.766 | 28.592 | 35.837 |
|  | OP | 30 | 35.200 | 7.151 | 1.306 | 32.530 | 37.870 |
|  | OO | 27 | 34.205 | 7.894 | 1.519 | 31.082 | 37.327 |
| **Post-MRI** | |  |  |  |  |  |  |
|  | PP | 29 | 30.240 | 5.395 | 1.002 | 28.188 | 32.292 |
|  | PO | 28 | 31.964 | 8.930 | 1.688 | 28.502 | 35.427 |
|  | OP | 30 | 32.570 | 5.620 | 1.026 | 30.472 | 34.669 |
|  | OO | 27 | 32.899 | 7.581 | 1.459 | 29.900 | 35.898 |

Supplementary Table 7. Relationship between administered substance and perception of substance received.

|  | | **Fear conditioning (Day 1)** | | |  | **Extinction learning (Day 2)** | | | | | | |
| --- | --- | --- | --- | --- | --- | --- | --- | --- | --- | --- | --- | --- |
|  | | Substance perceived on Day 1 | | |  | Substance perceived on Day 2 | | |  | Substance perceived on Day 1 | | |
|  | | Placebo | Oxytocin | Total |  | Placebo | Oxytocin | Total |  | Placebo | Oxytocin | Total |
| Substance administered | |  |  |  |  |  |  |  |  |  |  |  |
|  | Placebo | 39 | 19 | 58 |  | 34 | 25 | 59 |  | 35 | 23 | 58 |
|  | Oxytocin | 37 | 22 | 59 |  | 32 | 24 | 56 |  | 32 | 25 | 57 |
| Total | | 76 | 41 | 117 |  | 66 | 49 | 115 |  | 67 | 48 | 115 |

Supplementary Table 8. Whole-brain analysis to compare the OP and PP groups for the contrast CS+ > CS- during extinction learning.

| Cluster-level | |  | Voxel-level | | |  | Peak voxel MNI | | |  | Region |
| --- | --- | --- | --- | --- | --- | --- | --- | --- | --- | --- | --- |
| Voxel count | *p*_FWE_ |  | *t* | *z*_max_ | *p_FWE_* |  | x | y | z |  |  |
| 24 | 0.533 |  | 5.077 | 4.774 | 0.024 |  | 39 | -9 | -17 |  | Right hippocampus |
| 84 | 0.040 |  | 5.013 | 4.720 | 0.030 |  | -42 | 3 | -4.5 |  | Left insula |
| 50 | 0.169 |  | 4.406 | 4.199 | 0.212 |  | 51 | -3 | -19.5 |  | Right middle temporal gyrus |
|  |  |  | 3.826 | 3.685 | 0.746 |  | 51 | 9 | -17 |  |  |
|  |  |  | 3.711 | 3.582 | 0.848 |  | 48 | 3 | -12 |  |  |
| 22 | 0.579 |  | 4.343 | 4.145 | 0.252 |  | 45 | -51 | -24.5 |  | Right angular gyrus |
| 16 | 0.727 |  | 4.211 | 4.029 | 0.355 |  | 15 | -9 | 70.5 |  | Right superior frontal gyrus |
| 24 | 0.533 |  | 3.930 | 3.779 | 0.639 |  | -12 | -69 | -27 |  | Left cerebellum crus 1 |
|  |  |  | 3.260 | 3.170 | 0.997 |  | -6 | -66 | -22 |  | Left cerebellum lobule III |
| 10 | 0.872 |  | 3.851 | 3.708 | 0.721 |  | 6 | -39 | -14.5 |  | Vermis III |
| 28 | 0.450 |  | 3.748 | 3.615 | 0.818 |  | -60 | -57 | 5.5 |  | Left middle temporal gyrus |
|  |  |  | 3.506 | 3.396 | 0.961 |  | -66 | -54 | -2 |  |  |
| 12 | 0.826 |  | 3.725 | 3.594 | 0.837 |  | 60 | -48 | 8 |  | Left middle temporal gyrus |
| 30 | 0.412 |  | 3.614 | 3.494 | 0.913 |  | -18 | -72 | -12 |  | Left cerebellum lobule VI |
|  |  |  | 3.605 | 3.486 | 0.918 |  | -18 | -63 | -9.5 |  |  |
| 18 | 0.676 |  | 3.591 | 3.473 | 0.925 |  | -48 | -39 | 50.5 |  | Left inferior parietal lobule |
| 23 | 0.556 |  | 3.577 | 3.460 | 0.933 |  | -48 | -72 | 0.5 |  | Left middle occipital gyrus |
|  |  |  | 3.529 | 3.417 | 0.953 |  | -39 | -57 | 3 |  |  |

Supplementary Table 9. Whole-brain analysis to compare the PO and PP groups for the contrast CS+ > CS- during extinction learning.

| Cluster-level | |  | Voxel-level | | |  | Peak voxel MNI | | |  | Region |
| --- | --- | --- | --- | --- | --- | --- | --- | --- | --- | --- | --- |
| Voxel count | *p*_FWE_ |  | *t* | *z*_max_ | *p_FWE_* |  | x | y | z |  |  |
| 91 | 0.030 |  | 4.519 | 4.298 | 0.152 |  | -54 | -57 | 5.5 |  | Left middle temporal gyrus |
|  |  |  | 3.387 | 3.286 | 0.987 |  | -42 | -57 | 3 |  |  |
| 83 | 0.041 |  | 4.248 | 4.061 | 0.324 |  | -57 | -33 | 45.5 |  | Left inferior parietal lobule |
|  |  |  | 4.177 | 3.998 | 0.386 |  | -48 | -39 | 50.5 |  |  |
| 90 | 0.031 |  | 4.171 | 3.994 | 0.391 |  | 57 | -9 | 0.5 |  | Right superior temporal gyrus |
|  |  |  | 3.974 | 3.818 | 0.592 |  | 48 | -18 | 3 |  |  |
|  |  |  | 3.291 | 3.198 | 0.996 |  | 63 | -18 | 15.5 |  |  |
| 160 | 0.003 |  | 4.096 | 3.927 | 0.464 |  | 45 | -48 | 0.5 |  | Right middle temporal gyrus |
|  |  |  | 3.947 | 3.794 | 0.621 |  | 57 | -51 | 10.5 |  |  |
|  |  |  | 3.832 | 3.691 | 0.740 |  | 54 | -48 | -2 |  |  |
| 29 | 0.431 |  | 4.077 | 3.910 | 0.483 |  | -15 | -63 | -32 |  | Left cerebellum crus 1 |
|  |  |  | 3.765 | 3.631 | 0.803 |  | -9 | -78 | -42 |  |  |
| 25 | 0.511 |  | 4.067 | 3.901 | 0.494 |  | 3 | -57 | -17 |  | Vermis IV-V |
| 13 | 0.802 |  | 4.005 | 3.846 | 0.559 |  | -6 | 9 | -4.5 |  | Left caudate |
| 43 | 0.231 |  | 3.990 | 3.833 | 0.575 |  | 30 | -63 | 35.5 |  | Right middle occipital gyrus |
| 30 | 0.412 |  | 3.918 | 3.768 | 0.652 |  | 51 | 3 | -7 |  | Right temporal pole |
|  |  |  | 3.463 | 3.356 | 0.973 |  | 54 | 12 | -4.5 |  |  |
| 22 | 0.579 |  | 3.824 | 3.684 | 0.748 |  | 42 | -36 | 58 |  | Right postcentral gyrus |
|  |  |  | 3.206 | 3.120 | 0.999 |  | 33 | -36 | 65.5 |  |  |
| 12 | 0.826 |  | 3.818 | 3.679 | 0.753 |  | 48 | 12 | -17 |  | Right temporal pole |
| 12 | 0.826 |  | 3.807 | 3.669 | 0.764 |  | -36 | 3 | -9.5 |  | Left insula |
|  |  |  | 3.386 | 3.285 | 0.987 |  | -42 | 0 | -2 |  |  |
| 22 | 0.579 |  | 3.772 | 3.637 | 0.797 |  | 54 | -3 | -19.5 |  | Right middle temporal gyrus |
|  |  |  | 3.384 | 3.283 | 0.988 |  | 57 | -9 | -14.5 |  |  |
| 18 | 0.676 |  | 3.747 | 3.614 | 0.819 |  | 15 | -51 | 65.5 |  | Right superior parietal lobule |
| 15 | 0.752 |  | 3.744 | 3.612 | 0.821 |  | 51 | 33 | -7 |  | Right inferior frontal gyrus |
|  |  |  | 3.638 | 3.516 | 0.899 |  | 39 | 39 | -2 |  |  |
| 19 | 0.651 |  | 3.670 | 3.544 | 0.878 |  | 39 | 3 | 50.5 |  | Right precentral gyrus |
| 12 | 0.826 |  | 3.631 | 3.510 | 0.903 |  | -27 | -60 | 43 |  | Left superior parietal lobule |
|  |  |  | 3.304 | 3.210 | 0.995 |  | -24 | -69 | 43 |  |  |
| 10 | 0.872 |  | 3.626 | 3.504 | 0.907 |  | -51 | -39 | 18 |  | Left superior temporal gyrus |
|  |  |  | 3.474 | 3.366 | 0.970 |  | -60 | -39 | 15.5 |  |  |
| 14 | 0.777 |  | 3.615 | 3.495 | 0.913 |  | -18 | -75 | 43 |  | Left superior occipital gyrus |
|  |  |  | 3.291 | 3.198 | 0.996 |  | -15 | -69 | 48 |  |  |

Supplementary Table 10. Whole-brain analysis to compare the OO and PP groups for the contrast CS+ > CS- during extinction learning.

| Cluster-level | |  | Voxel-level | | |  | Peak voxel MNI | | |  | Region |
| --- | --- | --- | --- | --- | --- | --- | --- | --- | --- | --- | --- |
| Voxel count | *p*_FWE_ |  | *t* | *z*_max_ | *p_FWE_* |  | x | y | z |  |  |
| 43 | 0.231 |  | 4.700 | 4.455 | 0.086 |  | 51 | 0 | -4.5 |  | Right superior temporal gyrus |
|  |  |  | 3.450 | 3.344 | 0.976 |  | 54 | 9 | -12 |  |  |
| 26 | 0.490 |  | 4.490 | 4.273 | 0.165 |  | -42 | 3 | -2 |  | Left insula |
| 59 | 0.113 |  | 4.197 | 4.017 | 0.368 |  | -42 | -57 | 3 |  | Left middle temporal gyrus |
|  |  |  | 3.790 | 3.654 | 0.780 |  | -54 | -57 | 3 |  |  |
| 28 | 0.450 |  | 4.181 | 4.002 | 0.382 |  | 9 | -57 | -4.5 |  | Right lingual gyrus |
|  |  |  | 3.410 | 3.308 | 0.984 |  | 9 | -45 | -14.5 |  |  |
| 76 | 0.055 |  | 4.083 | 3.915 | 0.477 |  | 51 | -51 | 3 |  | Right middle temporal gyrus |
|  |  |  | 3.796 | 3.659 | 0.775 |  | 51 | -63 | 10.5 |  |  |
|  |  |  | 3.556 | 3.441 | 0.942 |  | 45 | -60 | -9.5 |  |  |
| 32 | 0.378 |  | 4.073 | 3.906 | 0.488 |  | -60 | -42 | 20.5 |  | Left superior temporal gyrus |
| 20 | 0.627 |  | 4.029 | 3.867 | 0.533 |  | -3 | -48 | 55.5 |  | Left precuneus |
| 11 | 0.850 |  | 4.005 | 3.846 | 0.559 |  | -42 | -78 | 8 |  | Left middle occipital gyrus |
| 15 | 0.752 |  | 3.950 | 3.797 | 0.617 |  | -24 | -6 | 8 |  | Left putamen |
| 14 | 0.777 |  | 3.763 | 3.629 | 0.805 |  | -6 | 3 | 28 |  | Left anterior cingulate gyrus |
| 12 | 0.826 |  | 3.757 | 3.623 | 0.810 |  | -21 | 0 | 20.5 |  | Left caudate |
| 14 | 0.777 |  | 3.712 | 3.583 | 0.847 |  | 3 | -42 | 43 |  | Right precuneus |
|  |  |  | 3.557 | 3.442 | 0.941 |  | -9 | -42 | 45.5 |  |  |
| 13 | 0.802 |  | 3.417 | 3.314 | 0.983 |  | -36 | -57 | 53 |  | Left inferior parietal lobule |
|  |  |  | 3.352 | 3.254 | 0.991 |  | -42 | -45 | 50.5 |  |  |

Supplementary Table 11. Multiple regression analysis revealed a significant positive correlation between differential skin conductance response (SCR; CS+ minus CS-) and bilateral anterior insulae.

| Cluster-level | |  | Voxel-level | | |  | Peak voxel MNI | | |  | Region |
| --- | --- | --- | --- | --- | --- | --- | --- | --- | --- | --- | --- |
| Voxel count | *p*_FWE_ |  | *t* | *z*_max_ | *p_FWE_* |  | x | y | z |  |  |
| 142 | 0.004 |  | 5.344 | 4.960 | 0.011 |  | -33 | 21 | 3 |  | Left insula |
|  |  |  | 4.863 | 4.564 | 0.060 |  | -39 | 15 | 0.5 |  |  |
|  |  |  | 3.578 | 3.448 | 0.949 |  | -39 | 6 | -7 |  |  |
| 220 | 0.0003 |  | 4.925 | 4.615 | 0.049 |  | 39 | 12 | -4.5 |  | Right insula |
|  |  |  | 4.302 | 4.087 | 0.318 |  | 30 | 27 | -2 |  |  |
|  |  |  | 3.781 | 3.629 | 0.825 |  | 57 | 12 | -4.5 |  |  |
| 16 | 0.735 |  | 4.086 | 3.899 | 0.520 |  | -39 | -3 | 45.5 |  | Left precentral gyrus |
| 23 | 0.561 |  | 3.956 | 3.785 | 0.656 |  | -6 | 21 | 25.5 |  | Left anterior cingulate gyrus |
|  |  |  | 3.396 | 3.283 | 0.991 |  | -3 | 12 | 20.5 |  |  |
| 18 | 0.684 |  | 3.862 | 3.702 | 0.751 |  | 3 | 9 | 43 |  | Right middle cingulate gyrus |
| 16 | 0.735 |  | 3.828 | 3.671 | 0.784 |  | -63 | -30 | 23 |  | Left superior temporal gyrus |

# References

1. Spielberger C, Gorsuch R, Lushene R. STAI Manual for the State― Trait Anxiety lnventory. Consulting Psychologists Press, Inc, California; 1970.

2. Toth I, Neumann ID, Slattery DA. Central administration of oxytocin receptor ligands affects cued fear extinction in rats and mice in a timepoint-dependent manner. Psychopharmacology. 2012;223(2):149-58.

3. Penny WD, Friston KJ, Ashburner JT, Kiebel SJ, Nichols TE. Statistical parametric mapping: the analysis of functional brain images: Elsevier; 2011.

4. Whitfield-Gabrieli S, Nieto-Castanon A. Conn: a functional connectivity toolbox for correlated and anticorrelated brain networks. Brain connectivity. 2012;2(3):125-41.

5. Nieto-Castanon A. Handbook of functional connectivity magnetic resonance imaging methods in CONN: Hilbert Press; 2020.

6. Andersson JL, Hutton C, Ashburner J, Turner R, Friston K. Modeling geometric deformations in EPI time series. Neuroimage. 2001;13(5):903-19.

7. Friston KJ, Ashburner J, Frith CD, Poline JB, Heather JD, Frackowiak RS. Spatial registration and normalization of images. Human brain mapping. 1995;3(3):165-89.

8. Henson R, Buechel C, Josephs O, Friston K. The slice-timing problem in event-related fMRI. NeuroImage. 1999;9:125-.

9. Sladky R, Friston KJ, Tröstl J, Cunnington R, Moser E, Windischberger C. Slice-timing effects and their correction in functional MRI. Neuroimage. 2011;58(2):588-94.

10. Whitfield-Gabrieli S, Nieto-Castanon A, Ghosh S. Artifact detection tools (ART). Cambridge, MA Release Version. 2011;7(19):11.

11. Power JD, Mitra A, Laumann TO, Snyder AZ, Schlaggar BL, Petersen SE. Methods to detect, characterize, and remove motion artifact in resting state fMRI. Neuroimage. 2014;84:320-41.

12. Calhoun VD, Wager TD, Krishnan A, Rosch KS, Seymour KE, Nebel MB, et al. The impact of T1 versus EPI spatial normalization templates for fMRI data analyses. Wiley Online Library; 2017. Report No.: 1065-9471.

13. Ashburner J. A fast diffeomorphic image registration algorithm. Neuroimage. 2007;38(1):95-113.

14. Ashburner J, Friston KJ. Unified segmentation. Neuroimage. 2005;26(3):839-51.

15. Friston KJ, Williams S, Howard R, Frackowiak RS, Turner R. Movement‐related effects in fMRI time‐series. Magnetic resonance in medicine. 1996;35(3):346-55.

16. Hallquist MN, Hwang K, Luna B. The nuisance of nuisance regression: spectral misspecification in a common approach to resting-state fMRI preprocessing reintroduces noise and obscures functional connectivity. Neuroimage. 2013;82:208-25.

17. Behzadi Y, Restom K, Liau J, Liu TT. A component based noise correction method (CompCor) for BOLD and perfusion based fMRI. Neuroimage. 2007;37(1):90-101.

18. Chai XJ, Castañón AN, Öngür D, Whitfield-Gabrieli S. Anticorrelations in resting state networks without global signal regression. Neuroimage. 2012;59(2):1420-8.

19. Worsley KJ, Marrett S, Neelin P, Vandal AC, Friston KJ, Evans AC. A unified statistical approach for determining significant signals in images of cerebral activation. Human brain mapping. 1996;4(1):58-73.

20. Chumbley J, Worsley K, Flandin G, Friston K. Topological FDR for neuroimaging. Neuroimage. 2010;49(4):3057-64.
